# Craniotomy in Semi-sitting Position: A 4-year Single Institution Experience

**DOI:** 10.4274/TJAR.2025.251874

**Published:** 2026-02-09

**Authors:** Arunabha Karmakar, Muhammad Jaffar Khan, Ayten Saraçoğlu, Merve Ergenç, Mogahed Ismail Hassan Hussein, Mohammed Janish Orompurath, Kemal Tolga Saraçoğlu, Kishore Kumar Gangineni, Pawel Ratajczyk, Neeraj Kumar

**Affiliations:** 1 Hamad Medical Corporation, Department of Anaesthesiology, Intensive Care Unit and Perioperative Medicine, Doha, Qatar; 2 Qatar University College of Medicine, Department of Anaesthesiology, Doha, Qatar; 3 University of Health Sciences Türkiye, Süreyyapaşa Chest Diseases and Thoracic Surgery Training and Research Hospital, Department of Anesthesiology, İstanbul, Türkiye; 4 Hamad Medical Corporation, Department of Research Analyst and Bioinformatics, Doha, Qatar; 5 Medical University of Lodz, Department of Anaesthesiology and Intensive Therapy, Lodz, Poland

**Keywords:** Craniotomy, neuroanaesthesia, perioperative care, pneumocephalus, sitting position, venous air embolism

## Abstract

**Objective:**

We aimed to determine patient outcomes after craniotomies performed in semi-sitting position in our institution from 2019-2023. Primarily, we examined surgical and anaesthetic (clinical) outcomes. Secondarily, we evaluated any major complications that may have occurred.

**Methods:**

Hospital records from 2019-2023 were retrospectively reviewed for adult patients who underwent craniotomy in the sitting position. Individual charts were examined for intra- and postoperative events. The demographic and clinically important findings were tabulated using Excel spreadsheet. The dataset was descriptively analyzed, with quantitative data represented as mean ± standard deviation, and qualitative data as valid percentages from the total cohort. Parametric comparisons of sex vs. (length of intensive care unit and hospital stay) and anaesthesia duration (in minutes) were performed using Student’s t-test. A 95% confidence level was used to determine statistical significance. Analyses were performed using IBM SPSS^®^ Edition 22.

**Results:**

From 2019-2023, 10 patients underwent craniotomy in a sitting position. General anaesthesia was induced and maintained using an intravenous target-controlled infusion of remifentanil and propofol. Nine patients developed pneumocephalus, with one developing increased intracranial pressure. One patient had a significant venous air embolism with severe manifestations, including massive pleural effusion. All patients except one were extubated at the end of the surgery.

**Conclusion:**

Of the 10 craniotomies performed in the sitting position from 2019-2023, 90% were managed without major long-term sequelae. Although the sitting position for craniotomies is not without challenges, a dedicated and experienced team can manage complications and improve patient outcomes.

**Main Points**

• Craniotomy is rarely performed in the sitting position to facilitate surgical exposure and drainage of cerebrospinal fluid and blood.

• Patients aged 50 years and younger were primarily candidates for cerebellopontine angle tumor resection or pineal gland lesion surgeries in the semi-sitting position, whereas older patients typically underwent the procedure for posterior fossa metastatic tumors.

• Pneumocephalus and venous air embolism are two potentially significant complications that may arise when performing craniotomy in the sitting position.

• A dedicated and experienced neuroanaesthesia team can manage possible complications and improve patient outcomes.

## Introduction

The semi-sitting position facilitates the drainage of the cerebrospinal fluid and blood by gravity and provides improved surgical exposure and a wide and clean surgical field.^1^ Anatomical orientation and early decompression of the cisterna magna are additional advantages.^2^ This position has been used for years in surgeries using the supracerebellar approach. Moreover, better neurosurgical outcomes have also been reported. However, there is an ongoing debate on whether these benefits outweigh the risks to patients.^3^ Maintaining the patient’s head above heart level during surgery causes the intracranial venous pressure to reach a negative value. Air entry into the venous system can develop due to pressure changes and can lead to air embolism. Air can infiltrate the circulation through any open vein or dural sinus in the surgical region.^4^ The semi-sitting position poses a higher risk of venous air embolism (VAE) than the prone position.^5^ The frequency of air embolism is as high as 38.6% and has been reported to be more common in posterior fossa surgeries than in cervical spinal procedures.^6^

The neurosurgery team at our institution successfully performs more than 1000 surgeries annually while seeking to maintain high-quality care. As an academic center, we also have a neurosurgical residency program and a neuroanaesthesia fellowship program. Together with faculty-level physicians, experienced neurophysiologists and nurses, our institution provides a multidisciplinary team approach to deliver safe patient care. In a systematic manner, our neurosurgical and anaesthesia teams reassess their practice every few years as part of their self-appraisal. Therefore, we retrospectively studied all cases of neurosurgery performed in the sitting position. This study aimed to determine patient outcomes in a semi-sitting position over the past 4 years. Our primary aim was to quantify our experience in terms of surgical and anaesthetic (clinical) outcomes. Our secondary aim was to evaluate the major complications in the semi-sitting position.

## Methods

Following ethical approval from the Medical Research Committee of Hamad Medical Corporation (approval no.: MRC-04-23-578; date: 21 Aug 2023), an electronic medical record database was used to retrospectively examine the patient records. Adult patients who underwent craniotomies in the sitting position between January 2019 and June 2023 were included. Individual patient charts were used to evaluate significant intra- and postoperative events. All the critical and adverse events were recorded.

**Figure 1:**
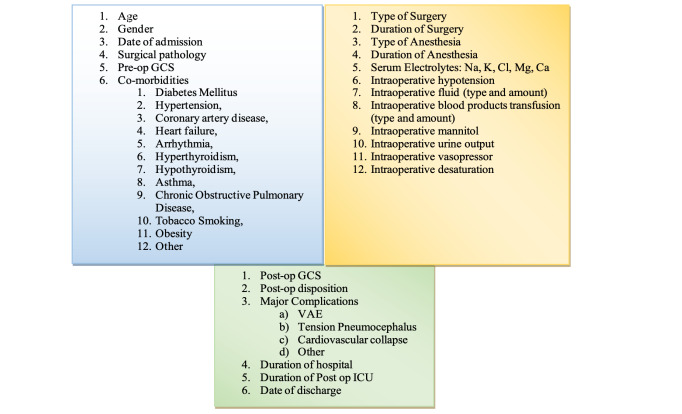
Data collection template. GCS, Glasgow Coma Scale; VAE, venous air embolism; ICU, intensive care unit.

The data collection template is shown in Figure 1. The data collected were de-identified and coded for the statistical analyses.

The study dataset was descriptively analyzed using quantitative data represented as mean ± standard deviation and qualitative data represented as valid percentages from the total cohort. A possible parametric comparison involving sex, length of intensive care unit (ICU) stay, hospital stay, and duration of anaesthesia (in minutes) was performed using Student’s t-test. A 95% confidence level was used to determine statistical significance. All analyses were performed using the IBM SPSS^®^  Edition 22.

## Results

Ten patients underwent craniotomies in the sitting position at our institution from January 2019 to June 2023. The patient demographics are presented in Table 1. No patients were excluded from the analysis.

### Baseline Characteristics

All patients had a preoperative Glasgow Coma Scale (GCS) score of 15 and normal preoperative echocardiography. Intraoperatively, all patients were administered target-controlled infusions of propofol and remifentanil intravenously. All patients also had an arterial line and a central venous catheter inserted along with a Foley catheter to monitor urine output.

The sex ratio was 1:1, with five males and five females. All patients were between 33 and 60 years of age. Of note, patients 50 years and younger underwent surgery for cerebellopontine angle (CPA) tumor resections or for lesions in the pineal gland. Two patients older than 50 years underwent surgery for metastatic tumors in the posterior fossa.

### Types of Surgeries

CPA tumor resection was the most common surgery (n = 5). Three surgeries were performed for lesions in the pineal region, and two for metastatic tumors in the posterior fossa. All surgeries lasted for an average of >7 hours (363-512 min; mean, 443 min; standard deviation, 49.6 min). All procedures were performed in a semi-sitting position, using Mayfield pins to fix the skull. All patients received general anaesthesia with endotracheal intubation using propofol and remifentanil target-controlled infusion modes for total intravenous anaesthesia and muscle relaxation. Almost all patients had post-induction hypotension and required intravenous vasopressors. Seven of 10 patients received a phenylephrine (PE) bolus or infusion; while all 10 received norepinephrine (NE) to maintain blood pressure. Nine patients had pneumocephalus, and one patient developed severe manifestations. One patient developed a clinically significant VAE with severe manifestations, including massive pleural effusion and adult respiratory distress syndrome. Except for this one patient, all patients were extubated at the end of surgery. A summary of the major complications is presented in Table 2.

**Figure 2:**
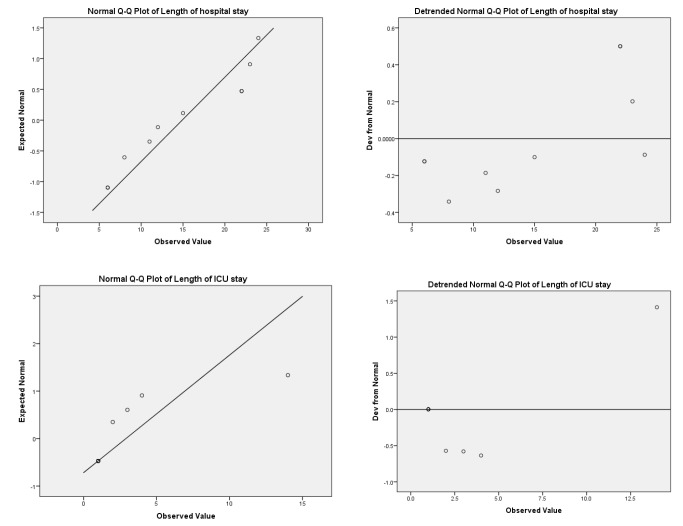
Normal and detrended normal Q-Q plots of the length of hospital and intensive care unit stay.

The detrended normal Q-Q plot depicted the differences between the observed and predicted hospital and ICU stays (Figure 2).

### Case Presentations

**Figure 3:**
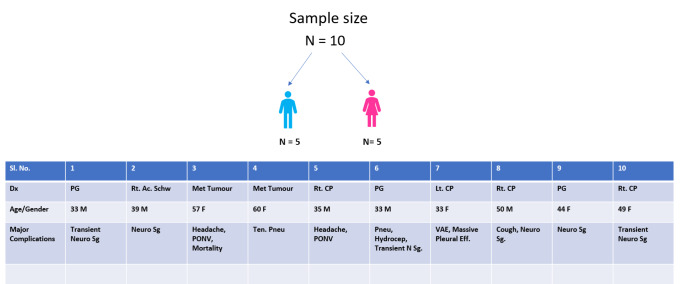
The distribution of case presentations. Dx, diagnosis; Rt, right; Lt, left; PG, pineal gland tumor; Ac. Schw, acoustic schwannoma; Met, metastatic; CP, cerebellopontine angle; Ten. Pneu, tension pneumocephalus; Pneu, pneumoncephalus; Hydrocep, hydrocephalus; N. Sg/Neuro Sg, neurological symptoms/signs; VAE, venous air embolism; PONV, postoperative nausea and vomiting; Eff., effusion.

The distribution of case presentations is summarized in Figure 3.

### Case 1 

A thirty-three-year-old male presented with pineal gland pilocytic astrocytoma and underwent left occipital craniotomy. The preoperative GCS score was 15. Known medical risks and comorbidities included hyponatremia. Preoperative echocardiography was normal. The duration of anaesthesia was 7 h and 55 min. Intraoperatively, he received 2100 mL of normal saline (NS), 500 mL of colloids, and 100 µg of PE, along with 35 g of mannitol. His urine output was 1500 mL, and blood loss was estimated at 250 mL. No VAE was detected during surgery, and pneumocephalus occurred without complications. Postoperatively, the patient experienced hyponatremia, possibly related to inappropriate antidiuretic hormone secretion or adrenal insufficiency. The patient also exhibited unequal pupils and upward-gaze palsy, which gradually improved over time.

### Case 2 

A thirty-nine-year-old male patient was diagnosed with right cerebellopontine (CP) angle acoustic schwannoma and underwent surgical intervention. The preoperative GCS score was 15. Known medical risks or comorbidities included hypertension, obesity, gastric ulcer, and lumbar spondylosis. Preoperative echocardiography was normal. The duration of anaesthesia for this complex procedure was 8 h and 32 min. Intraoperatively, the patient received 2300 mL of NS and 500 mL of colloids, while 292 µg of NE was given to maintain hemodynamic stability. Additionally, 50 g mannitol was administered, and the patient exhibited a urine output of 3000 mL. The estimated intraoperative blood loss was 300 mL. Notably, VAE was detected during the procedure but was managed successfully. Pneumocephalus occurred but resulted in no complications. Postoperatively, the patient experienced postoperative nausea and vomiting (PONV) and was extubated in the operating room. However, he developed hypertension immediately after surgery. He also exhibited lower motor neuron palsy in the right facial nerve.

### Case 3

A fifty-seven-year-old female patient with a history of invasive breast ductal carcinoma developed cerebellar metastasis to the left posterior fossa. The preoperative GCS score was 15. Known medical risks or comorbidities included hypertension, diabetes mellitus, obesity, and metastatic breast cancer. Preoperative echocardiography was normal. The surgery lasted for 6 h and 35 min. During the surgery, the patient received 1000 mL of NS, 500 mL of plasma protein fraction (PPF), 900 µg of PE, and 840 µg of NE to maintain hemodynamic stability. Additionally, 80 g of mannitol was administered, and the patient exhibited a urine output of 1500 mL, with an estimated blood loss of 250 mL. No VAE was detected during the procedure, and the patient recovered uneventfully in the operating room.

No immediate postoperative complications occurred. However, cranial pathology progressed, and the patient succumbed to the condition. 

### Case 4

A sixty-year-old female patient was diagnosed with metastatic cervical cancer that had spread to the left superior cerebellar peduncle. The preoperative GCS score was 15. Known medical risks or comorbidities included smoking tobacco, hypertension, and metastatic cervical cancer (metastases to the lungs and brain). Preoperative echocardiography was normal. The surgery lasted 6 h and 46 min. Intraoperative bradycardia episodes occurred during surgical manipulation. During the procedure, the patient received 1500 mL of NS, 500 µg of PE, and 0.3817 mg of NE to manage hemodynamic parameters. Specific details regarding plasma proteins or other administered substances were not provided. The estimated blood loss was 250 mL.

Significant postoperative complications were observed. The patient developed pneumocephalus in the subdural space of the left temporal and both frontal lobes, resulting in mass effect and increased intracranial pressure (ICP), which was associated with hypertension and bradycardia. Postoperatively, multiple episodes of nausea and vomiting were observed. Although the patient showed some improvement in preoperative cerebellar signs, she still exhibited an ataxic gait.

### Case 5 

A thirty-three-year-old male patient was diagnosed with right acoustic schwannoma. The preoperative GCS score was 15. Known medical risks or comorbidities included hypertension. Preoperative echocardiography was normal. The surgery lasted for 6 hours and 3 minutes. During the procedure, the patient received 500 mL of NS, 500 mL of PPF, and 1000 mL of plasmalyte. Hemodynamic stability was maintained with the administration of 466.6 µg of PE, 1291.66 µg of NE, and 12 mg of ephedrine. The estimated intraoperative blood loss was 250 mL.

Postoperatively, the patient did not experience any immediate complications, such as VAE or pneumocephalus. He reported only PONV. The patient did not require prolonged respiratory support. The absence of immediate complications and successful intraoperative management suggested a favorable outcome for this patient in the early postoperative period.

### Case 6 

A thirty-three-year-old male patient was diagnosed with a meningothelial meningioma, typically found in the meninges, which was detected near the pineal glands.

The preoperative GCS score was 15. The patient had no known medical comorbidities. Preoperative echocardiography was normal. The surgery lasted for 6 h and 56 min. During the procedure, the patient received 2000 mL of NS and 1000 mL of PPF. Hemodynamic stability was maintained with the administration of 513.3 µg of NE. Mannitol (40 g) was administered to manage ICP, and the patient exhibited a urine output of 2800 mL, with an estimated blood loss of 300 mL.

Postoperatively, the patient’s recovery was initially uneventful, with no immediate complications such as VAE or pneumocephalus. However, the patient developed dilation of the lateral and third ventricles and a right temporal subgaleal hematoma. This was associated with staring episodes; an external ventricular drain was inserted to manage increased ICP. The patient improved; however, he also experienced diplopia, left foot numbness, and later developed headaches.

### Case 7 

A thirty-three-year-old female patient was diagnosed with left CP angle vestibular schwannoma. The preoperative GCS score was 15. She had a history of obesity, obstructive sleep apnea, and hypertension. Preoperative echocardiography was normal. The surgery lasted for 7 h and 40 min. Intraoperatively, to manage fluid balance and hemodynamic stability, the patient received 2100 mL of NS, 500 mL of PPF, and 500 mL of Ringer’s lactate (RL). Additionally, 2.48 mg of NE was administered to maintain blood pressure. Mannitol (45 g) was administered during surgery, and the patient exhibited a urine output of 3000 mL, with an estimated blood loss of 100 mL.

However, the postoperative course was marked by significant complications related to VAE. VAE manifested during the surgery, leading to its diagnosis, with intraoperative episodes of desaturation and a decrease in end-tidal carbon dioxide (EtCO_2_). Postoperatively, the patient exhibited signs of VAE-induced pulmonary complications, including pulmonary congestion and high oxygen requirements. To manage these complications, furosemide was administered, and the patient was intubated for respiratory support. Subsequently, 100 mg of hydrocortisone was administered.

Despite these challenges, the immediate surgical recovery was uneventful. However, the patient's postoperative stay in the surgical ICU (SICU) was characterized by two additional episodes of VAE. These episodes were accompanied by persistent high oxygen requirements and the development of multiple complications, including acute respiratory distress syndrome, massive pleural effusion, acute kidney injury, and sepsis. The patient also experienced sick euthyroid syndrome, which was attributed to the VAE.

Upon further follow-up at the neurosurgery clinic, the patient presented with left-sided facial weakness that required continued evaluation and management.

### Case 8 

A fifty-year-old male patient was diagnosed with right CP angle schwannoma. The preoperative GCS score was 15. She also had a history of hypertension. The surgery lasted for 8 h and 31 min. Preoperative echocardiography was normal. During the procedure, the patient received 3500 mL of NS and 1250 mL of PPF to maintain fluid balance. Hemodynamic stability was maintained with the administration of 417 µg of PE and 1.44 mg of NE. Mannitol (50 g) was administered, and the patient exhibited a urine output of 1800 mL, with an estimated blood loss of 400 mL.

Postoperatively, the patient’s immediate surgical recovery was uneventful, with no complications during the intraoperative phase. However, during his hospital stay, the patient developed nosocomial meningitis, which required treatment. Fortunately, he responded well to the treatment and showed improvement. During long-term follow-up, the patient reported right-sided facial numbness, which may be a neurological consequence of the surgery or related complications.

### Case 9

A forty-four-year-old female patient was diagnosed with a large pineal parenchymal tumor with intermediate differentiation. The preoperative GCS score was 15. She was COVID-19 positive. Preoperative echocardiography was normal. The surgery lasted for 7 h and 45 min. During the procedure, the patient received 1200 mL of NS and 1000 mL of PPF for fluid management. Hemodynamic stability was maintained with the administration of 1000 µg of PE, 240 µg of NE, and 40 mg of labetalol (used to control one episode of surge in blood pressure exceeding 170 mmHg). Mannitol (30 g) was administered and the patient exhibited a urine output of 1600 mL, with an estimated blood loss of 250 mL.

The patient’s immediate postoperative surgical recovery was uneventful, with no complications noted during the intraoperative phase. However, during the postoperative period, the patient experienced occasional polyuria, horizontal nystagmus, on-and-off diplopia, and blurred vision in the left eye.

### Case 10 

A forty-nine-year-old female patient was diagnosed with a right vestibular schwannoma. She had a history of migraines. Preoperative echocardiography was normal. The surgery lasted for 7 h and 10 min. During the procedure, the patient received 500 mL of NS, 750 mL of PPF, and 500 mL of RL for fluid management. Hemodynamic stability was maintained with the administration of 660 µg of PE and 800 µg of NE. Additionally, mannitol (20 g) was administered, and the patient exhibited a urine output of 1800 mL, with an estimated blood loss of 200 mL.

The patient’s immediate postoperative surgical recovery was uneventful, with no complications noted during the intraoperative phase. However, during the neurosurgical follow-up, right-sided Bell’s palsy was observed.

## Discussion

In this case series, we analyzed the outcomes of 10 patients who underwent intracranial surgery in the semi-sitting position. Owing to the serious risks of VAE, hemodynamic instability, tension pneumocephalus, and quadriplegia, the semi-sitting position in craniotomy has long been a topic of debate in the fields of neurosurgery and neuroanaesthesia.

Our results indicated that patients aged 50 years and younger were primarily candidates for CP angle tumor resection or pineal gland lesion surgeries in the semi-sitting position, whereas older patients typically underwent the procedure for posterior fossa metastatic tumors. These findings align with the literature’s understanding that the choice of position often depends on specific surgical indications and patient characteristics.

Anaesthetic management is a critical factor in the success of craniotomies in a semi-sitting position. Our study highlights the need for vigilant hemodynamic control, as most patients require vasopressor support owing to post-induction hypotension. The incidence of hypotension in the sitting position ranges from 5% to 32%.^7^ This is theorized to be due to venous pooling in the legs caused by gravity, which leads to a reduced cardiac preload. This effect is further compounded by anaesthesia-mediated vasodilation and myocardial depression. Preloading patients with crystalloid solution before assuming a sitting position has been shown to reduce the incidence of hypotension.^8^ Our institution does not mandate preloading, although some practitioners do. Our patients were maintained on vasopressor therapy, commonly PE infusion or occasionally NE infusion, to regulate blood pressure, and received fluid therapy only to ensure euvolemia.

Pneumocephalus is a potential complication of semi-sitting craniotomy. This was a common occurrence in our cohort, with one patient experiencing severe manifestations. Importantly, we documented a case of clinically significant VAE, which is a rare but potentially life-threatening complication.

The existing literature on VAE presents a variety of data. Different monitoring techniques have been employed to diagnose VAE, and severity classifications have been used to categorize its varying levels. Additionally, it’s worth noting that most of these studies were retrospective in nature.^9^ Previous studies have reported varying EtCO_2_ thresholds for diagnosing clinically significant VAE.^10^ For example, Durmuş et al.^11^ established a limit of 5 mmHg. In contrast, Feigl et al.^12^ determined this limit to be 3 mmHg, whereas Ammirati et al.^13^ settled at 5 mmHg. In the present study, we did not set a specific EtCO_2_ threshold. Instead, we considered an unexplained reduction, accompanied by tachycardia and desaturation, in patients undergoing procedures in the sitting position to be indicative of a clinically significant VAE.

The incidence of VAE has also been reported in different publications. In a systematic review of 977 patients undergoing cervical spine or posterior fossa surgery in the semi-sitting position, air embolism occurred in 40.2% of the patients.^6^ In a case series reported by Kurihara and Nishimura,^14^ 26% of 23 patients, operated in the semi-sitting position, experienced VAE, with an EtCO_2_ reduction by more than 5 mmHg. A retrospective study by Ammirati et al.^13^ found that 26.8% of 41 patients had a decrease of >5 mmHg. In another study evaluating the risk of paradoxical VAE in patients with a patent foramen ovale, 9.6% exhibited an EtCO_2_ increase >3 mmHg.^12^ Finally, in a prospective study, the reported incidence rate was 4%.^15^

Head elevation is closely associated with the incidence of complications. Durmuş et al.^11^ employed a 40° head elevation and did not observe any clinically significant air embolism leading to EtCO_2_ reductions >5 mmHg or hemodynamic changes. In this case series, 11 patients underwent craniotomy in the dynamic lateral semi-sitting position between 2020 and 2022. Notably, none of these patients experienced clinically significant VAEs or major complications during surgery, and all were successfully extubated postoperatively. Postoperative imaging confirmed the total removal of the tumors and cavernomas, reflecting the efficacy of the surgical procedures. In contrast, our study involving 10 patients over 4 years of age revealed that while most patients were safely extubated, one patient suffered from a clinically significant VAE with severe manifestations. The use of medical anti-shock trousers or anti-gravity, to reduce the incidence of VAE has been described in the literature.^16, 17, 18^ Inflation of the trousers to 40 mmHg in the leg compartment and 30 mmHg in the abdominal compartment led to an increased right atrial pressure, which, in turn, linearly increased the pressure on the venous sinuses and thereby reduced the risk of VAE. However, this is not a widely accepted practice, and practitioners must consider peripheral vessel and nerve compression, as well as abdominal compression. This was not part of our institution’s routine practice.

These findings emphasize the complexity and potential risks associated with craniotomies in the sitting position and the importance of careful patient selection and anaesthesia management to ensure safe outcomes. In a prospective study, clinically significant air embolisms occurred in 8% of patients with 30° head elevation and 50% of patients with 45° head elevation.^4^ In our study, the incidence was 10% with 35° head elevation.

### Study Limitations

Our study was retrospective in nature and had a small sample size of ten patients. The critical findings reported in our study may not necessarily translate into other centers with dissimilar case volumes of craniotomy performed in the semi-sitting position. Nevertheless, we believe that the findings are relevant for reflecting standards and quality of management adopted for each individual case.

## Conclusion

In conclusion, our study contributes to the ongoing discussion on the use of the semi-sitting position for craniotomies. It emphasizes the complexity and potential risks associated with this position. It highlights the critical role of careful patient selection, anaesthesia management, and adherence to safety protocols to ensure optimal outcomes in neurosurgical procedures. It is essential to continue evaluating positioning techniques and monitoring methods to enhance patient safety and the quality of care in neurosurgical procedures.

## Ethics

**Ethics Committee Approval:** Following ethical approval from the Medical Research Committee of Hamad Medical Corporation (approval no.: MRC-04-23-578; date: 21 Aug 2023).

**Informed Consent:** An electronic medical record database was used to retrospectively examine the patient records.

## Figures and Tables

**Table 1. Demographics of Patients in a Semi-Sitting Position:** 

**Demographics**	**Range**	**Mean**	**Standard deviation**
Age (years)	33-60	43.30	10.27
Weight (kg)	60-137	85.26	23.17
BMI kg m^2^	24.2-49.6	30.31	8.47

BMI, body mass index

**Table 2. Major Complications and Their Frequency in Patients Who Underwent Craniotomy in the Semi-Sitting Position:** 

**Major hypotensive event**				
-	Frequency	Percent	Valid percent	Cumulative percent
Yes	9	90.0	90.0	90.0
No	1	10.0	10.0	100.0
Total	10	100.0	100.0	-
**Venous air embolism**				
-	Frequency	Percent	Valid percent	Cumulative percent
No	9	90.0	90.0	90.0
Yes	1	10.0	10.0	100.0
Total	10	100.0	100.0	-
**Pneumocephalus**				
-	Frequency	Percent	Valid percent	Cumulative percent
No	9	90.0	90.0	90.0
Yes	1	10.0	10.0	100.0
Total	10	100.0	100.0	-
**Delayed extubation**				
-	Frequency	Percent	Valid percent	Cumulative percent
No	9	90.0	90.0	90.0
Yes	1	10.0	10.0	100.0
Total	10	100.0	100.0	-
**Post-operative nausea and vomiting**				
-	Frequency	Percent	Valid percent	Cumulative percent
No	7	70.0	70.0	70.0
Yes	3	30.0	30.0	100.0
Total	10	100.0	100.0	-
